# Discovering Potential Drug Targets for Irritable Bowel Syndrome Through Genetic Insights: A Mendelian Randomization and Colocalization Study

**DOI:** 10.1155/grp/7800569

**Published:** 2025-10-30

**Authors:** Yitong Li, Yao Jiao, Muyuan Wang, Jiayan Hu, Yali Yuan, Yingdi Qu, Wenji Zhang, Yitong Yu, Xinyu Lu, Chengtao Liang, Zhengdao Lin, Junxiang Li, Tangyou Mao, Chune Xie

**Affiliations:** ^1^Dongfang Hospital, Beijing University of Chinese Medicine, Beijing, China; ^2^Shenzhen Bao'an Traditional Chinese Medicine Hospital, Shenzhen, China

**Keywords:** colocalization analysis, drug targets, genetics, irritable bowel syndrome, Mendelian randomization

## Abstract

**Background:**

Irritable bowel syndrome (IBS), a gastrointestinal motility disorder affecting millions of patients worldwide, has a substantial impact on healthcare economics and patient quality of life. However, fully satisfactory therapeutic options remain lacking. The identification of pathogenic proteins supported by causal genetic evidence enables the exploration of potential therapeutic targets for IBS.

**Methods:**

A Mendelian randomization (MR) study was performed to discover potential treatment targets linked to IBS. Summary data for IBS (outcome) were acquired from the two largest independent cohorts: sample sizes of 486,601 (53,400 cases and 433,201 controls) and 101,884 (24,735 cases and 77,149 controls), respectively. Instrumental variables were derived from cis-expression quantitative trait loci (cis-eQTL) data of druggable genes, obtained through the eQTLGen Consortium database. Colocalization analysis was employed to assess whether IBS risk and gene expression were influenced by shared SNPs. An IBS mouse model was additionally utilized to confirm the therapeutic potential of drug targets.

**Results:**

Four drug targets (*P2RY14*, *SLC5A6*, *ATRAID*, and *IL1RL1*) displayed notable MR findings in two separate datasets. Purinergic receptor P2Y14 (P2RY14) and all-trans retinoic acid–induced differentiation factor (ATRAID) exhibited robust evidence of colocalization with IBS. We further showed an abnormal increase in expression of P2RY14 and a significant decrease in ATRAID level in the colon tissue of IBS mice.

**Conclusion:**

This study proposes two potential therapeutic targets for IBS: P2RY14 and ATRAID. Drugs aimed at targeting these two genes have a greater chance of success in clinical trials, potentially facilitating the prioritization of IBS drug development and lowering associated costs.

## 1. Background

Irritable bowel syndrome (IBS), a prevalent stress-associated functional disorder of the gastrointestinal tract, manifests through a spectrum of enteric and neuropsychiatric symptoms including abdominal discomfort, altered bowel habits (diarrhea and/or constipation), and psychological disturbances [1]. Epidemiological surveys have found that the global incidence of IBS is about 3.8% [2]. According to reports, IBS accounts for 3.1 million outpatient visits per year in US healthcare facilities, and the total direct and indirect expenditures for the diagnosis and treatment of IBS exceed $20 billion, resulting in a significant socioeconomic burden [3]. However, due to the heterogeneity of the disease, the pathophysiological mechanism of IBS remains unclear. Genetic factors, immune factors, environmental influences, inflammatory and infectious agents, psychological factors, food hypersensitivity, and alterations in gut microbiota and intestinal permeability, all impact the brain–gut axis, thereby affecting gastrointestinal function and motility and triggering diarrhea-predominant IBS (IBS-D) [4]. Clinical treatment of IBS is mainly symptomatic treatment by regulating intestinal flora, antidiarrheal and spasmodic medications, antianxiety and depression treatments, and adjusting dietary habits, which has some limitations such as easy recurrence, more side effects, and no cure. Consequently, there is a pressing need to investigate the underlying mechanisms of IBS pathogenesis and discover novel therapeutic targets for this condition.

Although large-scale randomized controlled trials (RCTs) are often considered the gold standard for evaluating treatment strategies, there may be limitations in terms of feasibility, cost, and ethics. Recently, Mendelian randomization has emerged as a widely utilized approach for both drug repositioning and the identification of novel therapeutic interventions [5]. Drug efficacy can be predicted through simulated RCT [6, 7]. Genome-wide association studies (GWASs) have detected particular single-nucleotide polymorphisms (SNPs) located on chromosomes that control protein expression, with these genetic variants being designated as protein quantitative trait loci (pQTL). Cis-acting variants are genetic tools at the proximal end of transcriptional gene units, located on the same chromosome as transcriptional genes, which make it easier to identify functional variants associated with phenotypes, thereby directly revealing genetic mechanisms that control the regulation of gene expression [8, 9]. By employing MR analysis, SNP–gene expression data and SNP-disease associations can be combined to determine causal relationships between exposures and outcomes, enabling the systematic screening of drug targets and biomarkers.

During zygote formation, genetic inheritance follows the principle of random segregation of chromosomes, which mirrors the fundamental design of RCTs, where participants are randomly assigned to different groups to minimize bias. After fertilization, people are randomly assigned to high or low expression levels of drug-available gene variants, and individuals often do not know their genotype, so Mendelian randomization studies are similar to blind trials. Mendelian randomization minimizes the confusion and bias of reverse causality common in traditional observational studies and eliminates the interference of multiple confounding factors [10].

This investigation presents novel insights within the IBS research domain, focusing on the discovery of therapeutic targets for disease progression modulation. We contrasted the identified expression quantitative trait loci (eQTLs) with data from two separate case–control studies of IBS for MR analysis. In addition, we performed a colocalization analysis to rule out potential confounders and investigate promising therapeutic targets for the condition.

## 2. Methods

### 2.1. Ethical Approval and Study Design

**Figure 1:**
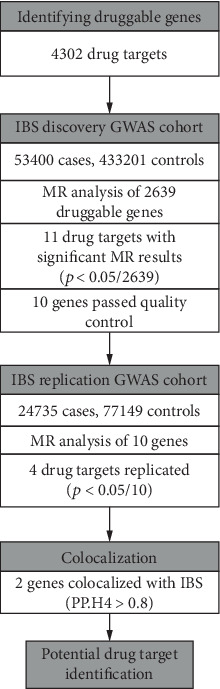
Overview of the study design in our Mendelian randomization study.

Our investigation constitutes a secondary analysis of publicly accessible data, adhering to rigorous standards of scientific inquiry. Within the original GWAS protocol, all participants had provided informed consent, ensuring the ethical integrity of the primary data collection. Given that our study solely leverages aggregated statistics derived from these pre-existing datasets, no additional ethical clearance is deemed necessary. The methodology and research process design employed in this endeavor are concisely illustrated in Figure 1, offering a transparent overview of our analytical approach.

### 2.2. Exposure Data

Human genes encoding molecular targets for therapeutic agents constitute what is known as the druggable genome. This includes approved and clinically developed protein therapeutic targets, proteins linked to drug targets and compounds, as well as proteins that belong to key drug target families.

The compilation of pharmacologically targetable genes was derived from a previous study. Within this investigation, a comprehensive tally of 4302 genes, each assigned a Human Gene Nomenclature Committee (HGNC) identifier, was discerned on the autosomes [11]. It is considered that the closer eQTLs are to genes of interest in drug development research, the more direct the regulatory effect on gene expression becomes. For the development of genetic instruments corresponding to all 4302 therapeutic targets, cis-eQTLs located within a 100 kb genomic window surrounding each gene's position were systematically selected. Ultimately, eQTLs for 2639 druggable genes were obtained.

### 2.3. Outcome Data

GWAS data for the IBS discovery cohort was acquired from prior research conducted on individuals of European ancestry [12]. This study included 53,400 IBS cases and 433,201 controls from a cohort with European ancestry. These cases of IBS must fulfill at least one criterion from the following four: (1) fulfill the Rome III symptom criteria outlined in the Digestive Health Questionnaire (DHQ), without necessitating alternative diagnostic interpretations for these symptoms; (2) possess a DHQ self-reported or electronic health record documenting a prior IBS diagnosis; (3) voluntarily provide a “self-reported diagnosis”; (4) indicate a self-reported IBS diagnosis when asked, “has a medical professional ever informed you of any… serious health conditions?” Hospital admission statistics reveal that IBS was either the primary or secondary ICD-10 diagnosis. For comprehensive data details, please refer to the original literature [11].

For consistency, GWAS data from 24,735 Rome III IBS patients and 77,149 controls from European lineages were selected for external replication [13]. The original dataset encompassed two separate European populations: the UK Biobank and the Lifelines cohort study.

### 2.4. Mendelian Randomization Analysis

We used the TwoSampleMR R package for MR analysis. We loaded and coordinated the exposure and outcome data with built-in functionality. The genetic instrumental variables for MR analysis are subject to three MR assumptions: Assumption 1: These SNPs must be strongly associated with exposure. Assumption 2: These SNPs have an effect on the outcome only through exposure. Assumption 3: These SNPs are not associated with confounding factors [14]. Therefore, some quality control was carried out in this study to filter out low-quality genetic instruments before MR analysis was performed. In order to minimize potential bias arising from weak instrument effects, SNPs with *F*-statistic less than 10 [*F* = (beta/se)^2^] were systematically excluded from the analysis [15], and we chose SNPs that were conditionally independent and free from linkage disequilibrium (*r*^2^ < 0.1, according to the European reference panel of the 1000 Genomes Project) as instrumental variables (Tables S1 and S2). Finally, we employed the Steiger filtering technique to eliminate genes with more SNP interpretation result (IBS) variation than exposed variation.

For the main analysis, we derived MR estimates for individual SNPs through the application of the Wald ratio method and meta-analyzed SNP estimates using inverse variance weighting (IVW), MR-Egger, and weighted median models with multiple proposed instruments. The assumption underlying IVW is that all genetic instruments are valid, and when this assumption holds true, IVW offers the greatest statistical power among the available methods [16]. Where more than two SNPs were available for each exposure, we examined whether the MR-Egger intercept deviated markedly from zero, thereby accounting for any potential pleiotropic influences in the relationship between the exposure in question and the outcome (judged at *p* < 0.05). For the sensitivity analyses, significance thresholds were adjusted for multiple testing using Bonferroni's method to establish appropriate corrected values. Within the discovery cohort, statistical significance was defined as *p* values less than 1.89e − 5 (*p* = 0.05/2639). Significant genes that passed quality control were repeatedly validated in the IBS replication GWAS cohort, with associations yielding a *p* value of less than 0.005 (*p* = 0.05/10) deemed statistically significant.

### 2.5. Colocalization Analysis

For significant MR results from two related cohorts, we performed a colocalization analysis of IBS risk using coloc R packages with default priors [17]. We synchronized the exposure and outcome datasets utilizing the “TwoSampleMR” package. We used default priors: *p*1 = 1e − 4, *p*2 = 1e − 4, and *p*12 = 1e − 5. *p*1, *p*2, and *p*12 are predetermined probabilities that SNPs in the test region are substantially associated with IBS risk, gene expression, or both. The colocalization analysis provides posterior probabilities for five potential hypotheses: PP.H0 indicates no link to either trait, PP.H1 suggests an association with gene expression but not the IBS trait, PP.H2 implies an association with the IBS trait alone, PP.H3 indicates an association with the IBS trait and expression of the gene, with distinct causal variants, and PP.H4 suggests an association with the IBS trait and expression of the gene, with a shared causal variant [16]. A threshold of PP.H4 > 0.80 was established for colocalization significance, enabling the identification of genes colocalized with IBS as potential drug targets.

### 2.6. Animal Experiment and IBS Modeling

C57BL/6 male mice were provided by SPF (Beijing) Biotechnology Co. Ltd. (Beijing, China). All mice were housed in a specific pathogen-free animal room maintained at a temperature of 20°C–24°C, with a humidity of 50%–60%, and a controlled light/dark cycle of 12 h each. The mice had free access to food and sterile water at all times. The IBS-D model was induced using neonatal maternal separation (NMS) combined with restraint stress (RS). The induction protocol was as follows: During the postpartum period from Days 4 to 21, neonatal mice were separated from their lactating mothers for 3 h daily, from 8:00 to 11:00 a.m. On Day 22, the mice were weaned to complete the separation between mothers and infants. From Days 22 to 49, the mice were fed a normal diet and water. At the age of 50–59 days, the mice underwent RS stimulation for 2 h each day, from 9:00 to 11:00 a.m. Following this stress regimen, the IBS-D mouse model was established.

Three mice were selected from the normal control group (no treatment), and another three mice that had been treated with NMS and RS were also chosen. Subsequently, euthanasia was performed on the mice. The mice were anesthetized with isoflurane using an anesthesia machine, with the flow rate adjusted to 0.5 L per minute. The animals were then placed in an induction chamber. Upon achieving a state of deep anesthesia, they were euthanized through cardiac puncture for blood collection, maintaining an anesthesia concentration of 1.5% during the process. Following euthanasia, colonic tissues were collected for further analysis. All experimental procedures followed the recommendations of the National Institutes of Health (NIH) for the Care and Use of Laboratory Animals and were cleared by the Animal Welfare and Ethics Review Board of Beijing Medconnex Biotech Co. Ltd. (Approval No. MDKN-2024-023).

### 2.7. Western Blotting for P2RY14 and ATRAID Expression in IBS Mice

Mouse colon tissues were lysed in RIPA buffer, and whole-cell proteins were extracted. The protein concentration was determined using the bicinchoninic acid (BCA) method. The protein solution was mixed with 5× reduced protein loading buffer at a ratio of 4:1, denatured in a metal bath at 95°C for 10 min, and then subjected to SDS-PAGE electrophoresis. Following electrophoresis, the proteins were transferred to a PVDF membrane and blocked with 5% skimmed milk. The prepared primary antibody was added, and the membrane was incubated overnight at 4°C. Subsequently, each membrane was washed three times for 5 min each with an appropriate wash buffer and then incubated with the secondary antibody for 30 min. Standard procedures were followed to initiate chemiluminescence, with ImageJ software (NIH) utilized for quantifying protein band intensities.

### 2.8. Evaluation of Druggability and Clinical Development Activity

We conducted an exhaustive search in databases such as DrugBank (http://go.drugbank.com), ChEMBL (http://www.ebi.ac.uk), and the Therapeutic Target Database (http://db.idrblab.net/ttd/) to evaluate the potential druggability of these genes by seeking mutations linked to phenotypic abnormalities. This endeavor is aimed at discovering potential repurposing candidates for drugs that could act on the druggable gene targets identified through MR (presumably referring to Mendelian randomization or a similar method) and colocalization analysis.

## 3. Results

### 3.1. Discovery Analysis

Utilizing cis-eQTL data sourced from the eQTLGen Consortium, we identified 2639 potential druggable genes following clustering and subsequently conducted a two-sample MR analysis on pooled European statistics from patients with IBS. Within the discovery cohort, comprising 53,400 IBS patients and 433,201 controls, we employed an IVW meta-analysis to integrate the effect estimates for each genetic instrument. Our findings in the discovery cohort revealed a causal link between the expression of 11 genes and the risk of IBS, with statistical significance at *p* < 1.89e − 5 (*p* < 1.89e − 5 = 0.05/2639, Bonferroni correction for 2639 drug targets; Table S3). However, the *BLK* gene failed to satisfy the horizontal pleiotropy test (*p* < 0.05; Table S4), thus necessitating its exclusion from subsequent analyses.

### 3.2. Replication Analysis

**Figure 2:**
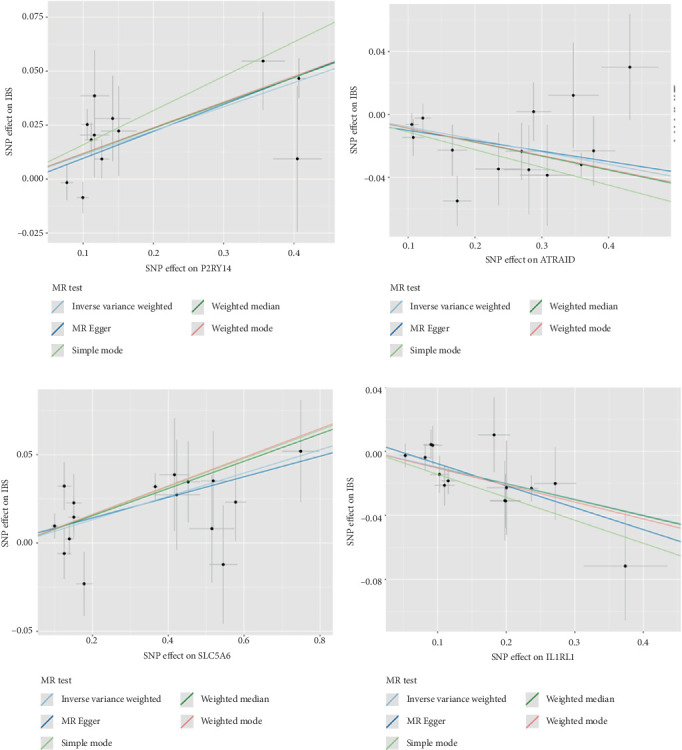
Scatter plot of the associations of four genes and the risk of irritable bowel syndrome. (a) Scatter plot of the analysis for P2RY14. (b) Scatter plot of the analysis for ATRAID. (c) Scatter plot of the analysis for SLC5A6. (d) Scatter plot of the analysis for IL1RL1.

During the replication stage, the research employed an additional GWAS dataset comprising 24,735 IBS cases and 77,149 European ancestry controls. The MR analysis was conducted similarly to that of the discovery cohort. We aimed to replicate the significance of all genes that reached significance in the discovery phase within the IBS replication GWAS cohort, utilizing either the Wald ratio or IVW method. Four drug targets (*P2RY14*, *SLC5A6*, *ATRAID*, and *IL1RL1*) surpassed a stringent Bonferroni-corrected significance threshold of *p* < 0.005 (*p* < 0.005 = 0.05/10 genes, Tables 1 and 2, Figure 2; Table S5). The horizontal pleiotropy assessment showed no notable pleiotropic impacts for these four genes (Table S6).

### 3.3. Colocalization Analysis

**Figure 3:**
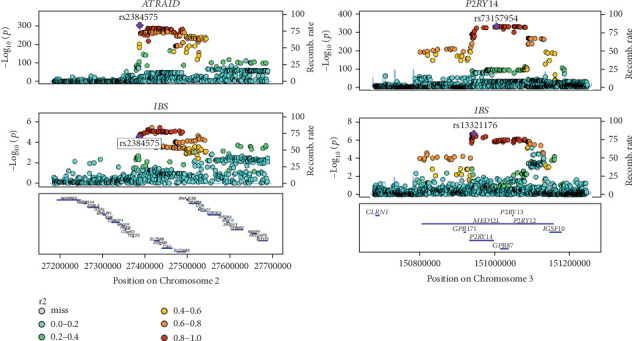
Regional Manhattan plot of associations of SNPs with P2RY14 and ATRAID locus.

When SNPs are distinctly linked to both the exposure and the outcome, colocalization analysis can be utilized to determine if the same causal SNPs underlie both. Consequently, we performed a colocalization analysis to further confirm the probability that SNPs linked to IBS and eQTLs have common causal genetic factors. Ultimately, two potentially pharmacologically targetable genes, P2RY14 (PP.H4 = 0.917) and ATRAID (PP.H4 = 0.860), were identified as having a common genetic effect between eQTLs and IBS risk (Figure 3). The findings of the colocalization analysis for all four potential therapeutic target genes are presented in Table 3.

### 3.4. Significant Changes in ATRAID and P2RY14 Expression in IBS-D Mice

**Figure 4:**
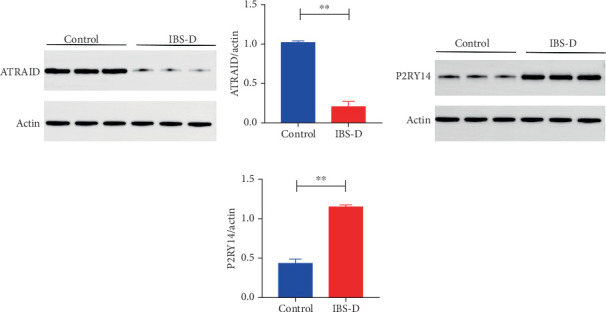
(a) Expression levels of ATRAID protein. (b) Quantitative analysis of ATRAID protein band intensity. (c) Expression levels of P2RY14 protein. (d) Quantitative analysis of P2RY14 protein band intensity. The double asterisks (∗∗) signify a statistically significant result with a *p* value of less than 0.01.

To investigate the potential relationship between the identified genes and the risk of IBS, we examined the protein levels of ATRAID and P2RY14 in the colon tissue of mice exhibiting IBS-D. Our findings revealed that the expression of ATRAID was significantly downregulated in IBS-D mice, whereas the expression of P2RY14 was significantly upregulated in these mice (Figure 4; the full-length gel and imprint are included.

### 3.5. Druggability of Identified Genes

We searched drug databases to pinpoint potential therapeutic targets for IBS using MR analysis and colocalization assessments. Currently, the number of molecular compounds targeting P2RY14 and ATRAID is limited (Table 4). MC-1, which targets the P2Y Purinoceptor 14, is currently being evaluated in clinical trials for coronary artery disease. Promethazine, a potential P2RY14 inhibitor, has been approved for use in allergic disorders, nausea, and vomiting. To date, no molecular compounds specifically targeting ATRAID have been discovered. In existing research, neither of these genes has been identified as specific drug targets for IBS.

## 4. Discussion

Our research employs extensive genome-wide MR and colocalization analyses to clarify the causal links between genes and IBS, providing insightful perspectives for the identification of novel therapeutic approaches. This study identified four potential drug targets for IBS: *P2RY14*, *SLC5A6*, *ATRAID*, and *IL1RL1*, based on several MR methods, including IVW, MR-Egger, weighted median, weighted mode, simple mode, and a horizontal pleiotropy test. However, due to linkage disequilibrium, some genes were omitted from the subsequent colocalization analyses. Ultimately, we recognized two genes that exhibit high levels of colocalization and have potential as new therapeutic targets for IBS. Our results indicate that both a decrease in P2RY14 expression and an increase in ATRAID expression are significantly associated with a reduced risk of IBS.

The P2Y14 receptor, also known as GPR105, belongs to the superfamily of G protein–coupled receptors and is broadly expressed in diverse cellular, tissue, and organ systems throughout the body. The P2Y14 receptor is highly prevalent in human adipose tissue, intestine, stomach, heart, and other tissues, where it is activated by UDP-sugar, an important extracellular signaling molecule. It comprises seven hydrophobic transmembrane helices interconnected via three extracellular and three intracellular loops [18]. Numerous studies pertaining to P2Y14R have emphasized its proinflammatory role in immune cells [19, 20], while the expression of P2Y14R has also been observed in epithelial, endothelial, and fibroblast cells [21, 22]. Studies have shown that purinergic receptors play a role in the development of gastrointestinal disorders and are currently under exploration as possible therapeutic interventions [23]. In mucosal biopsy samples obtained from patients with ulcerative colitis (UC), the expression level of P2Y14R was positively correlated with the severity of inflammation, indicating that P2Y14R may play a role in the regulation of inflammation. The knockout of P2Y14R has been shown to reduce the severity of colitis by reversing the necrotic apoptosis process in colonic tissue of mice with UC [24]. The P2Y14R found in intestinal epithelial cells (IECs) holds a crucial position in modulating the necrotic apoptosis of IECs and the inflammation associated with dextran sulfate sodium (DSS)–induced colitis. Furthermore, a novel P2Y14 receptor antagonist, characterized by high activity, high selectivity, and good pharmacokinetic properties, has demonstrated significant anti-inflammatory activity in a DSS-induced colitis model [25]. These findings underscore the importance of developing drugs that target the P2Y14 receptor.

The notion of a UDP-G/P2Y14R axis is gaining recognition due to the unique interaction between UDP-G and P2Y14R. The UDP-G/P2Y14R signaling cascade functions as a pivotal proinflammatory agent across various systems, thus emerging as a promising therapeutic target for anti-inflammatory strategies [26]. In addition, P2Y14R is expressed on neurons and also functions through glial cells [27, 28]. Recent research has additionally indicated that P2Y14R may play a role in neuropathic and inflammatory pain processes within the nervous system [29, 30]. In the trigeminal ganglion, P2Y14R facilitates orofacial pain by modulating the activation of satellite glial cells (SGCs), promoting the release of cytokines/chemokines such as IL-1*β*, TNF-*α*, and CCL2, as well as inducing phosphorylation of ERK1/2 and p38 MAPK [31]. IL-1*β* is a typical multifunctional proinflammatory cytokine that plays an important role in chronic pain, such as inflammatory pain, visceral pain, and neuropathic pain [32]. IL-1*β* causes neuronal pain sensitization primarily by increasing neuronal excitability [33, 34]. Visceral paresthesia and low-grade inflammation are also linked to the etiology of IBS. Our research revealed that P2Y14R plays a crucial role in the development of IBS, with elevated expression levels of P2Y14R observed in the colonic tissue of mice with IBS-D. Therefore, it has been hypothesized that inhibition of P2Y14R could be an effective treatment for IBS.

In colocalization analysis, ATRAID emerges as another pharmacologically relevant gene that surpasses the threshold of significance. Recently, ATRAID has been recognized as a gene linked to programmed cell death, also known as apoptosis-related Protein 3 (APR3). Nonetheless, the role of APR3 is still largely unknown. Attention has been drawn to the function of APR3 in apoptosis and cancer, with ongoing studies dedicated to uncovering its regulatory mechanisms within the context of oncogenesis [35, 36]. In terms of its structure, APR3 possesses binding domains for AP1, SP1, and MEF2D, hinting that nuclear regulators like nuclear factor of activated T cells (NFAT) and nuclear factor *κ*B (NF-*κ*B) could potentially serve as transcriptional activators for this gene [37]. Functionally, APR3 is implicated in apoptosis by inducing mitochondrial damage and releasing Cytochrome c from mitochondria [38]. Additionally, APR3 regulates the cell cycle by modulating the expression levels of Cyclin D1 [39], which in turn affects the progression and expansion of cancerous tumors and may facilitate cellular differentiation. Research has revealed that APR3 could hold a key position in modulating both apoptosis and autophagy processes, along with preserving the integrity of lysosomal membranes [40]. Autophagy is a process whereby substances within cells are transported to lysosomes for degradation following stimulation. This mechanism is vital in numerous physiological functions, encompassing metabolic challenges and the removal of detrimental substances [41]. It constitutes a cellular protection mechanism that safeguards cells exposed to stressful environments, such as toxins and metabolic stressors. In the scenario of elevated APR3 protein expression, there is an increase in lysosomal membrane permeability, accompanied by a notable drop in lysosomal pH [36]. Additionally, enzymes like LAP, CTSB, LAL, and *β*-gal exhibit heightened activities, suggesting that the APR3 protein facilitates lysosomal degradation and enhances phagocytic processes [37]. A recent study [42] has revealed that ATRA has the capability to elicit autophagy in APL cells through the inhibition of mTOR, implying that APR3 might induce autophagy through the ATRA signaling pathway. Furthermore, autophagy has been implicated in the development of IBS, where it has been reported to degrade the tight junction (TJ) protein claudin-2 via lysosomes, selectively reducing the permeability of TJ-associated ions and small molecules in epithelial cells [43]. Autophagy enhances the barrier function of IECs. Its mechanism is manifested through the degradation of components of the intestinal mucosal barrier by IECs via autophagy, as well as the immune response triggered by a large number of inflammatory mediators and xenoantigens interacting with intestinal mucosal-associated inflammatory cells [44]. Our study confirms that ATRAID levels are inversely associated with IBS risk. Furthermore, we analyzed the protein abundance of APR3 in mouse colon tissue and discovered a notable decrease in APR3 expression within the IBS-D mouse model. This finding indicates a potential correlation between APR3 levels and the risk of developing IBS. Currently, there are no molecular compounds specifically targeting ATRAID, and it is anticipated that this study will inspire further research into the role of APR3 in IBS.

This research offers several notable benefits. Firstly, it marks the inaugural identification of drug targets for IBS through the application of Mendelian randomization, utilizing an extensive dataset derived from GWAS on IBS risk. Additionally, this study replicated MR results in two large cohorts, substantially reducing the likelihood of false positives for genes achieving significance in both cohorts and thereby enhancing their potential as drug target genes. Secondly, we focused our efforts on druggable genes and identified two IBS-associated drug targets: P2RY14 and ATRAID. Thirdly, we employed colocalization analysis to prevent gene chain imbalances and provide a robust tool for pinpointing potential therapeutic targets. Furthermore, animal studies confirmed that these two proteins, P2RY14 and APR3, are also associated with IBS risk, bolstering confidence in the causal relationships. However, our study is not without limitations. Firstly, the ethnic homogeneity of the study cohort restricts the applicability of our findings to a broader range of ethnicities. As large-scale IBS GWAS become available in Asian populations, we plan to conduct transethnic comparisons to evaluate the consistency of our results. Secondly, we did not analyze systemic side effects, and this aspect requires further investigation. Lastly, it is imperative to conduct subsequent experimental validations and clinical trials to assess the therapeutic efficacy and safety of the identified targets in the treatment of IBS.

In summary, our study has successfully demonstrated a causal link between IBS and particular genes through rigorous whole-genome Mendelian randomization and colocalization analyses. This study has led to the identification of two promising drug targets for IBS therapy: P2RY14 and ATRAID. However, it is crucial to acknowledge that this study does not definitively establish their clinical effectiveness. Therefore, further experimental verification and clinical trials are essential to ascertain the therapeutic promise of the identified targets.

## Figures and Tables

**Table 1:** Mendelian randomization results in the discovery cohort.

**Genes**	**IBS discovery GWAS cohort**				
	**SNPs**	**OR (95% CI)**	**SNPs**	**MR-Egger intercept**	**SNPs**
P2RY14	12	P2RY14	12	P2RY14	12
SLC5A6	16	SLC5A6	16	SLC5A6	16
ATRAID	14	ATRAID	14	ATRAID	14
IL1RL1	14	IL1RL1	14	IL1RL1	14

**Table 2:** Mendelian randomization results in the replication cohort.

**Genes**	**IBS replication GWAS cohort**				
	**SNPs**	**OR (95% CI)**	**IVW ** **p** ** val**	**MR-Egger intercept**	**MR-Egger ** **p** ** val**
P2RY14	9	1.11 (1.04–1.17)	5.86e−04	0.005	0.614
SLC5A6	12	1.10 (1.05–1.15)	3.22e−05	−0.001	0.929
ATRAID	12	0.89 (0.85–0.93)	2.23e−06	−0.005	0.681
IL1RL1	11	0.87 (0.80–0.94)	2.18e−04	−0.003	0.820

**Table 3:** Colocalization results of eQTLs for four genes with IBS-associated SNPs.

**Genes**	**PP.H0**	**PP.H1**	**PP.H2**	**PP.H3**	**PP.H4**
P2RY14	0.000	0.001	0.000	0.082	0.917
SLC5A6	0.000	0.233	0.000	0.726	0.040
ATRAID	0.000	0.034	0.000	0.106	0.860
IL1RL1	0.000	0.687	0.000	0.151	0.162

**Table 4:** Identification of druggable targets.

**Gene**	**Target name**		**Drug name**	**Indication/associated conditions**
P2RY14	P2Y Purinoceptor 14	Investigational	MRS2690	Discovery agent
		In clinical trials	MC-1	Coronary artery disease
		Approved, investigational	Promethazine	Allergic conditions, nausea and vomiting, and motion sickness

ATRAID	Apoptosis-related Protein 3	Druggable	NA	NA

## Data Availability

All genome-wide association study (GWAS) datasets analyzed in this study are publicly available from the GWAS Catalog hosted by the European Bioinformatics Institute (EBI) (https://www.ebi.ac.uk/gwas/), under Accession Numbers GCST90016564 and GCST90243958. The datasets used and analyzed during the current study are available from the corresponding authors on reasonable request.

## Nomenclature

IBS: irritable bowel syndrome
MR: Mendelian randomization
RCT: randomized controlled trial
GWAS: genome-wide association studies
SNP: single-nucleotide polymorphism
pQTLs: protein quantitative trait loci
DHQ: Digestive Health Questionnaire
IVW: inverse variance weighting
NMS: neonatal maternal separation
RS: restraint stress
NIH: National Institutes of Health
BCA: bicinchoninic acid
UC: ulcerative colitis
IEC: intestinal epithelial cell

## Supporting Information

Supporting Information Additional supporting information can be found online in the Supporting Information section. The *R*^2^ values and *F*-statistics are presented in Tables S1 and S2. Table S3: Mendelian randomization analysis results in IBS discovery cohort. Table S4: The results of pleiotropy analysis in IBS discovery cohort. Table S5: Mendelian randomization analysis results in IBS replication cohort. Table S6: The results of pleiotropy analysis in IBS replication cohort.
